# Case report: Migratory biliary stent resulting in sigmoid colon perforation

**DOI:** 10.1093/jscr/rjae737

**Published:** 2024-11-24

**Authors:** Jasmine B Beloy, Nicholas P Lund, Annika M Van Hell, Shyam Allamaneni

**Affiliations:** Department of Surgery, The Jewish Hospital - Mercy Health, 4777 East Galbraith Rd, Cincinnati, OH 45236, United States; Department of Surgery, The Jewish Hospital - Mercy Health, 4777 East Galbraith Rd, Cincinnati, OH 45236, United States; Saba University School of Medicine, 27 Jackson Rd, Devens, MA 01434, United States; Department of Surgery, The Jewish Hospital - Mercy Health, 4777 East Galbraith Rd, Cincinnati, OH 45236, United States

**Keywords:** biliary stent, migration, perforation, biliary obstruction

## Abstract

Biliary stent insertion during endoscopic retrograde cholangiopancreatography is a therapeutic intervention to relieve obstruction and facilitate flow through the biliary tree. In rare circumstances, these stents can migrate and result in distal gastrointestinal perforation, which may necessitate endoscopic or surgical intervention. We report a case involving a 79-year-old female who presented with peritonitis due to sigmoid colon perforation following biliary stent migration. The stent was placed to treat acute cholangitis with choledocholithiasis. Two weeks following stent placement, gastroenterology attempted scheduled stent removal, but was unable to visualize the stent on endoscopy. Eleven days later, the patient was emergently taken to the operating room for an exploratory laparotomy and a Hartmann’s procedure for stent migration and subsequent sigmoid perforation. No established protocol exists for managing migratory biliary stents to avoid perforations. We emphasize the need for follow-up imaging and individualized clinical decision-making based on patient stability.

## Introduction

The utilization of stents to establish patency between the biliary tract and duodenum proves efficacious in various biliary conditions. Biliary stents come in metal or plastic variants [1]. While metal stents offer the advantage of infrequent occlusion, plastic stents are generally considered more cost-effective but with higher rates of perforation [1, 2]. Generally considered a safe and well-tolerated therapeutic intervention, biliary stents incur some risks. The most frequently reported complications are occlusion (11%) and bile leak (2.2%) [3]. The rate of stent migration varies among studies between ~1% and 10% [3, 4]. With ~37 reported cases of distant biliary stent migration and subsequent bowel perforation, we present a case involving a 79-year-old female with a history of diverticulosis who experienced migration and perforation of the sigmoid colon following biliary stent placement for acute cholangitis and choledocholithiasis [5].

## Case report

**Figure 1:**
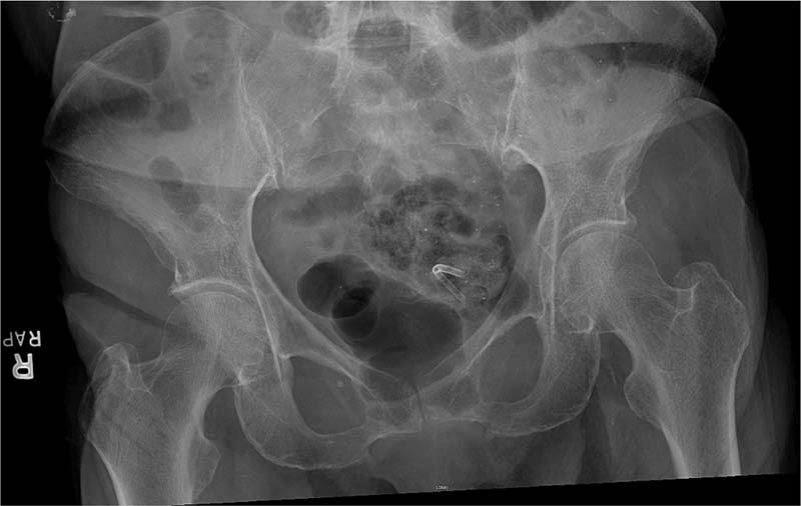
Abdominal X-ray of plastic biliary stent overlying the pelvis.

A 79-year-old female presented to the emergency department with progressively worsening abdominal pain, nausea, vomiting, intermittent chills, and non-bloody mucoid bowel movements. Her medical history was significant for a previous cardiovascular accident, dementia, emphysema, hypertension, gastroesophageal reflux disease, diverticulosis, type 2 diabetes mellitus, and hypothyroidism. The patient’s surgical history included a recent endoscopic retrograde cholangiopancreatography with biliary sphincterotomy, debris removal, and stent placement for acute cholangitis with choledocholithiasis. At that time, an 8.5 Fr × 7 cm straight plastic biliary stent was inserted in the common bile duct followed by a robotic-assisted laparoscopic cholecystectomy during the same admission. Two weeks later, an outpatient esophagogastroduodenoscopy (EGD) was performed for the removal of the biliary stent; however, it was not visualized protruding from the major duodenal papilla. Abdominal and pelvic radiographs revealed the migrated stent overlying the pelvis within the region of the sigmoid colon (Fig. 1). Given her stable clinical presentation, gastroenterology decided to wait for the stent to spontaneously pass.

**Figure 2:**
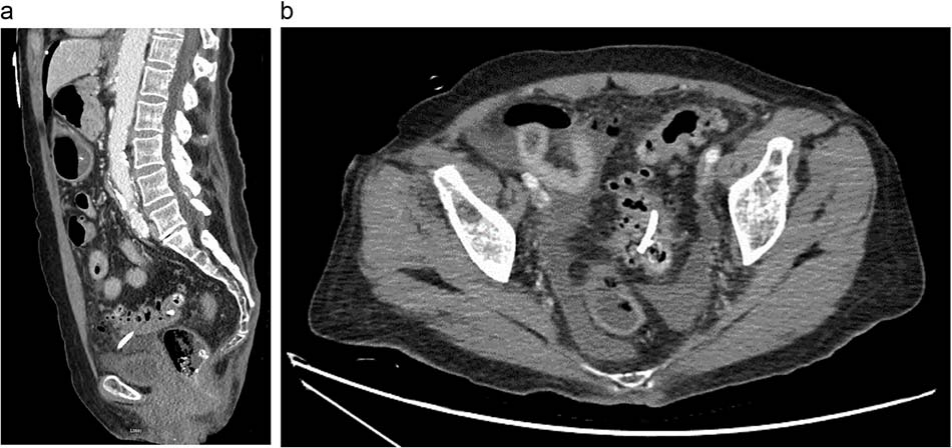
Abdominal CT showing migrated biliary stent that perforates the sigmoid colon in the sagittal (a) and axial (b) views. Pneumoperitoneum and free fluid in the pelvis are also noted.

Eleven days later, the patient presented to the emergency department with abdominal pain. During transit to the hospital, she exhibited mild hypotension with a systolic blood pressure in the 90s and was given 500 cc of intravenous normal saline. Upon arrival, her vital signs had stabilized with a temperature of 36.4°C, blood pressure of 122/63 mmHg, heart rate of 81 bpm and oxygen saturation of 94% on room air. Physical examination revealed a moderately distended, rigid abdomen with diffuse tenderness and involuntary guarding. Biochemical investigations demonstrated a leukocyte count of 4.2 x 10^9^ K/µL and a lactate of 3.2 mmol/L. Liver function tests, coagulation profile and electrolytes were unremarkable. Computed tomography (CT) imaging of the abdomen and pelvis with intravenous contrast demonstrated free fluid in the peritoneal cavity with moderate scattered pneumoperitoneum and migrated biliary stent that extended from the sigmoid colon into the peritoneal cavity (Fig. 2).

**Figure 3:**
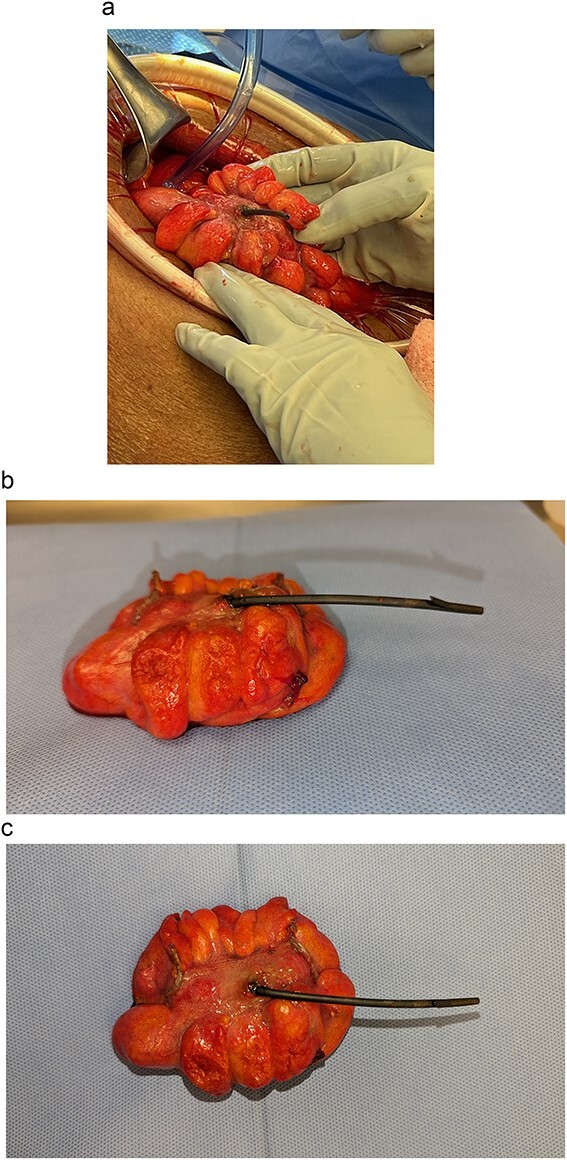
Migrated biliary stent perforating through a portion of sigmoid colon (a) intraoperatively and (b, c) post-resection.

Given the acute abdominal exam and imaging findings, the patient was emergently taken to the operating room. Diagnostic laparoscopy was quickly converted to exploratory laparotomy as gross stool encountered. The precise area of sigmoid colon perforation was identified (Fig. 3). We then proceeded with a Hartmann’s procedure (proctosigmoidectomy with end colostomy) with resection of the affected sigmoid colon. Her postoperative course was uneventful, and she was discharged to a skilled nursing facility. The pathology report of the resected sigmoid colon revealed prominent diverticular disease with inflammatory changes and an associated perforation. At her 2-month follow-up, the patient was tolerating her diet with a functioning colostomy.

## Discussion

Endoscopic biliary stents are a widely used modality to facilitate bile flow into the duodenum. Stents are equipped with flaps at their ends to mitigate migration risk; however, migration still occurs in ~5%–10% of patients [4]. Migrated stents can subsequently cause fistulas, abscesses, obstructions, and gastrointestinal perforations [5]. Diverticular disease increases the risk of perforation, with the sigmoid colon being the most common site [5]. The most common presenting symptom is abdominal pain [5].

Our patient received a biliary stent for the treatment of acute cholangitis followed by a robotic cholecystectomy. Upon discharge, plans were made for the stent’s retrieval in two weeks. EGD retrieval proved unsuccessful as the stent was not visualized, indicating probable migration. This was confirmed by a pelvic radiograph. Conservative management was pursued by gastroenterology with anticipation that the stent would spontaneously pass. The patient was discharged without any follow-up imaging. Days later, the patient returned with an acute abdomen requiring emergency surgery.

There is currently no established protocol for the management of migratory biliary stents to avoid perforation. In the rare cases of documented migrated stents, surgical intervention is used more often than endoscopic retrieval [5]. Clinical judgment regarding the nuances surrounding a patient’s age, comorbidities, and prognosis must be considered when deciding whether to opt for watchful waiting versus endoscopic retrieval or surgical intervention. Mortality rates associated with stent migration-induced intestinal perforation underscores the gravity of timely intervention.

There is still a staggering overall 30% mortality rate with up to 70% mortality rate observed in patients presenting with peritoneal signs [6]. Consequently, patients with generalized peritonitis require expeditious surgical intervention, as exemplified by our patient’s clinical trajectory.

We propose that patients being managed conservatively should be followed up with radiographic imaging to confirm stent passage per rectum. Imaging would allow clinicians to opt for endoscopic retrieval if failure to pass occurs. Although rare, our case emphasizes the importance of serial imaging of migrated stents to prevent subsequent perforation.
